# Electrical Distribution of Heat, Light, and Power

**Published:** 1889-09

**Authors:** 


### Electrical Distribution of Heat, Light, and Power. By Harold P. Brown, Electrical Engineer. New York, 1889: Press of J. W. Pratt & Son, 73 to 79 Fulton Street.

This pamphlet of about forty-eight pages, is an address before the International Medico-Legal Congress in New York, June 4th, 1889, protesting against dangerous methods of transmitting vast amounts of electrical energy through dense populations for industrial purposes, heating, and lighting. It is, therefore, particularly interesting to physicians. The author says:

"If the near future is to see a thousand electrical horse-power distributed where now we have but one, it is clearly the physician's duty to point out the dangerous currents, and it remains for the lawyer to secure wise legislative action preventing the adoption of systems or apparatus which needlessly jeopardize human life or health."

There are two principal methods or systems of generating and conveying electrical currents of great power. One is the continuous current flowing steadily in one direction, which can be rendered perfectly safe up to a pressure of fifteen hundred volts, and the other the alternating current, which flows first in one direction and then back again, the changes back and forth being made many thousand times in a second. Such a current as this develops an energy that overcomes all obstacles of insulation, and strikes for the shortest circuit. If that circuit be a human body, it is instant death. It is for all *practical* purposes a Faradic or secondary current of frightful power.

Mr. Brown vehemently protests against the use of such currents, and proposes a law forbidding their use except at exceedingly low pressures. This brings him in collision with a great corporation using the alternating current system—The Westinghouse Electric Light and Power Company, controlled by the famous Mr. Westinghouse, the inventor of the wonderful air-brake now used on all railroads.

Mr. Westinghouse denies the allegations made by Mr. Brown of danger in such currents, but fails to accept Mr. Brown's challenge to try it upon his own person. Physicians who have used the ordinary Faradic coil well know what a strong impression can be made upon the nervous system from one of the most feeble of these machines, with its insignificant battery of but a single volt; and we have ourselves seen a spark six inches long from a large Rhumkorff coil, actuated by a single battery cell, penetrate a bundle of several plates of window glass. From this small experience we are of opinion that the truth is on Mr. Brown's side. However, his excellent essay makes this sufficiently clear to any unprejudiced reader. W. M. J.

